# Effects of the Chinese traditional fitness practice Wuqinxi on balance improvement in older women with a history of falls: a randomized controlled trial

**DOI:** 10.3389/fpubh.2025.1503309

**Published:** 2025-03-06

**Authors:** Yutao Jiang, Heng Liu

**Affiliations:** ^1^BAYI Orthopedic Hospital, China RongTong Medical Healthcare Group Co. Ltd., Chengdu, China; ^2^Affiliated Sport Hospital of Chengdu Sport University, Chengdu, China; ^3^College of Physical Education, Chongqing University, Chongqing, China

**Keywords:** balance training, older adult health, fall risk reduction, gender-based interventions, randomized trial

## Abstract

**Objective:**

This study evaluated the impact of Wuqinxi Qigong, a traditional Chinese fitness practice, on the balance abilities of older women with a history of falls.

**Methods:**

Participants in the experimental group (*n* = 35) and the control group (*n* = 36), matched for age, height, and weight, engaged in a 24-week Wuqinxi exercise program (three times per week, 70 min per session). Dynamic and static balance abilities were assessed at weeks 0, 12, and 24.

**Results:**

Within the experimental group, compared to baseline, the movement distance of the center of pressure with open eyes (left and right) decreased by 17.0 and 22.1% at weeks 12 and 24, respectively (*p* < 0.05). The movement distance with closed eyes, the total length of displacement of the center of pressure, and the speed of center of pressure (left and right) decreased by 17.1, 8.6, and 16.6% at week 24 (*p* < 0.05). The one-leg stand time with eyes open and closed increased by 47.7, 68.0, and 77.1%, 80.6% at weeks 12 and 24, respectively (*p* < 0.01). Compared to week 12, the one-leg stand time with eyes open increased by 19.9% at week 24 (p < 0.01).

**Conclusion:**

A 24-week Wuqinxi exercise regimen enhances both static and dynamic balance abilities in older women with a history of falls. A longer regimen further improves static balance with eyes open compared to the 12-week mark.

## Introduction

Balance ability is an essential fundamental function required to perform daily activities, such as standing or walking (1, 2). Human balance ability is correlated with age, being relatively stable between 21 and 50 years, and gradually declines with increasing age, with the most significant decline occurring after the age of 70, thereby increasing the risk of falls (3, 4). A longitudinal study of 5,131 men and women (ages 40 to 95) indicated that women are more prone to falls. This gender disparity may be attributed to lower muscle mass, higher rates of osteoporosis (5). According to a report from the U.S. Centers for Disease Control and Prevention, approximately 33% of older individuals aged 65 to 75 experience falls annually (with a history of falls), and about 50% of those with a history of falls will fall again (6). It is reported that in China, among the older population aged 65 and above, about 21% of men and 43% of women have a history of falls (7). Falls can lead to soft tissue injuries (8), joint dislocations (9), fractures (10), and even head injuries (11), imposing a significant financial burden on families in terms of medical expenses. Therefore, improving the balance ability of the older to prevent falls is a topic worth attention. Studies have shown that regular physical activity can increase the balance ability of the older, such as regular Tai Chi (12, 13), resistance training (14, 15), and aerobic fitness exercises (16, 17).

Wuqinxi, a traditional Chinese exercise mimicking five animal movements (those of the tiger, bear, deer, ape, and bird), was created by Hua Tuo over 1800 years ago (18, 19). It integrates dynamic postures, breathing techniques, and mindfulness. Similar to dance-based interventions, it combines physical challenges with cognitive engagement, potentially enhancing neuroplasticity and balance control (20). Previous studies have shown that regular practice of Wuqinxi has a positive impact on the balance ability of healthy older individuals (21–25). The limitations of these studies are mainly in the following aspects: First, the subjects of the studies are mostly healthy older, and there is a lack of literature on the older with a history of falls. Second, existing studies rarely distinguish whether the static or dynamic balance ability of the older is improved after practicing Wuqinxi. Additionally, the relationship between exercise time and effect is not clearly defined, and whether the duration of exercise affects the improvement of balance ability has not been fully verified.

Given these gaps, this study aims to explore the impact of Wuqinxi exercise on balance ability by conducting a 24-week exercise regimen for older women with a history of falls, using both cross-sectional and longitudinal control methods. This will provide a reference for designing Wuqinxi exercise plans of different durations and enrich the theory of increasing balance ability and preventing falls in the older. The research hypothesis is that Wuqinxi exercise has a positive effect on improving the balance ability of older women with a history of falls, and that the effect of balance ability improvement is better with longer intervention time.

## Materials and methods

### Participants

This study was approved by the ethics committee of Chengdu Sport University (No: 202306). Leveraging the public fitness activities organized by the Sichuan Province Qigong Association, older women with a history of falls within the past year were recruited through the distribution of questionnaires and face-to-face interviews. Inclusion criteria were as follows: Female, aged between 60 and 70 years; At least one accidental fall within the past year; Undergone a physical examination for cardiac function; Have the intention to exercise and are able to complete the entire experimental process; In compliance with the Declaration of Helsinki and have signed an informed consent form. Exclusion criteria included: Regular exercise habits through other means within the past 6 months; Foot deformity, abnormal gait; A history of severe lower limb trauma; Epilepsy, motor disorders; Cardiovascular diseases.

**Figure 1:**
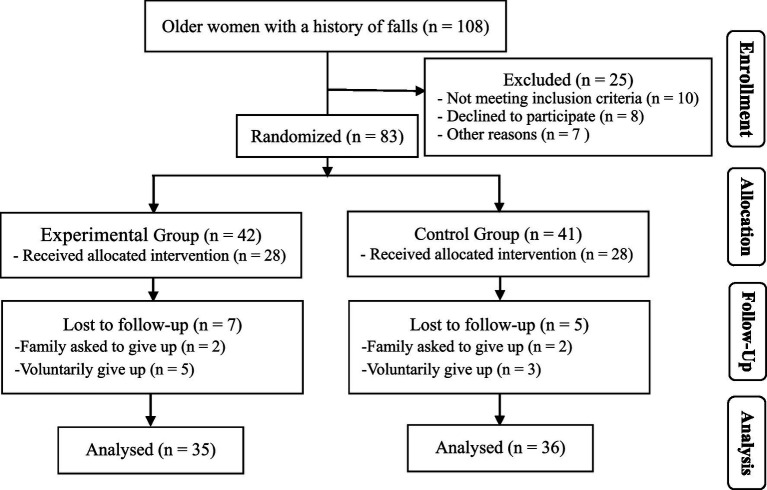
Participant selection flow diagram.

Based on previous studies (21–25) and considering a 3 (number of measurements) x 2 (number of groups) experimental design, it was anticipated that the Wuqinxi exercise would increase the one-leg standing time of older women with a history of falls by an average of 70%. The calculation was performed using G*Power software, with a significance level of 0.05 and a statistical power of 0.8. Accounting for approximately 15% sample attrition, at least 80 subjects were calculated to be necessary. At the beginning of the experiment, 83 participants were recruited. Participants were stratified by age, height, and weight, and then randomly assigned to the experimental and control groups using a computer-generated random sequence. During the process, 12 participants left due to personal reasons, resulting in a 14.5% sample loss, and ultimately 71 participants completed the entire process (35 in the experimental group and 36 in the control group, Figure 1; Table 1). There was no statistically significant difference in baseline data between the groups (*p* > 0.05).

**Table 1:** Demographic characteristics of the participants at baseline.

Index		Experimental group (*n* = 35)	Control group (*n* = 36)
Age (years)		65.4 ± 4.3	65.2 ± 3.6
Height (cm)		160.2 ± 7.2	159.3 ± 6.3
Weight (kg)		62.7 ± 5.1	62.9 ± 4.9
Distance of movement of the centre of pressure during standing with eyes open (mm)	Left and Right	90.6 ± 37.4	91.2 ± 21.6
	Front and back	170.6 ± 39.8	166.0 ± 34.2
Distance of movement of the centre of pressure during standing with eyes closed (mm)	Left and Right	94.3 ± 30.3	95.8 ± 25.7
	Front and back	230.6 ± 69.5	233.8 ± 73.0
One-leg standing time with eyes (s)	Open	15.3 ± 4.2	16.2 ± 5.7
	Close	10.3 ± 3.6	11.5 ± 6.2
Total length of displacement of the centre of pressure (mm)		1320.2 ± 266.3	1294.1 ± 218.2
Speed of movement of the centre of pressure (mm/s)	Left and Right	46.3 ± 11.0	45.0 ± 16.0
	Front and back	52.3 ± 16.2	51.8 ± 12.6

This study adopted a single-blinding design. To minimize potential biases, data analysts were blinded to group allocation, and participants were informed that the study aimed to evaluate ‘general fitness programs’ to minimize expectation bias. Screening procedures were standardized and conducted at the same time of day for all participants to ensure consistency.

### Practice of the Wuqinxi exercise

The Wuqinxi exercise regimen was conducted under the guidance of a professional Wuqinxi instructor, where the experimental group practiced the five animal forms of the exercise, which include the Tiger, Deer, Bear, Ape, and Bird forms. The exercise period was 24 weeks, with the first 4 weeks designated as the learning phase and weeks 5 to 24 as the consolidation and reinforcement phase. The exercise frequency and duration were determined based on prior studies demonstrating optimal adherence and physiological adaptation in older adults (22, 23). The frequency of exercise was three times per week, with each session lasting 70 min, including 10 min for warm-up and relaxation activities. Participants practiced to fixed voice prompts and musical rhythms, completing four full sets of Wuqinxi movements, each set taking approximately 14 min with a 1-min interval in between. Following the research design of Cheng et al. (26, 27), after completing each set of movements, participants immediately pressed on the radial artery to test the pulse beats for 10 s and reported their heart rate per minute to the experimenters. The exercise intensity was controlled within the range of (220 - age) x (55 to 65%). Participants were allowed to take a break if their heart rate was too high. Additionally, without changing their original lifestyle habits, weekly follow-ups were conducted by the experimenters via telephone or face-to-face interviews to ensure that the participants did not engage in other forms of regular fitness activities.

Compliance with the exercise regimen was managed through the following methods: regular supervision, with professional staff overseeing the training to ensure participants followed the set plan; completion of training logs, requiring participants to record detailed information about each training session, including content, duration, and intensity; and regular follow-ups, maintaining contact with participants through phone calls, WeChat, or face-to-face meetings to understand their training status and provide necessary support.

### Balance ability testing

The balance ability tests were conducted on three occasions: at baseline, at the 12th week, and at the 24th week. This study selected the Good Balance testing device to assess static and dynamic balance abilities because it has demonstrated good reliability and validity in previous studies and can precisely measure the movement distance and speed of the center of pressure (28). Both static and dynamic balance tests were conducted with participants standing on the test platform using a stopwatch to measure the time that participants could stand on one leg with eyes open and closed.

**Figure 2:**
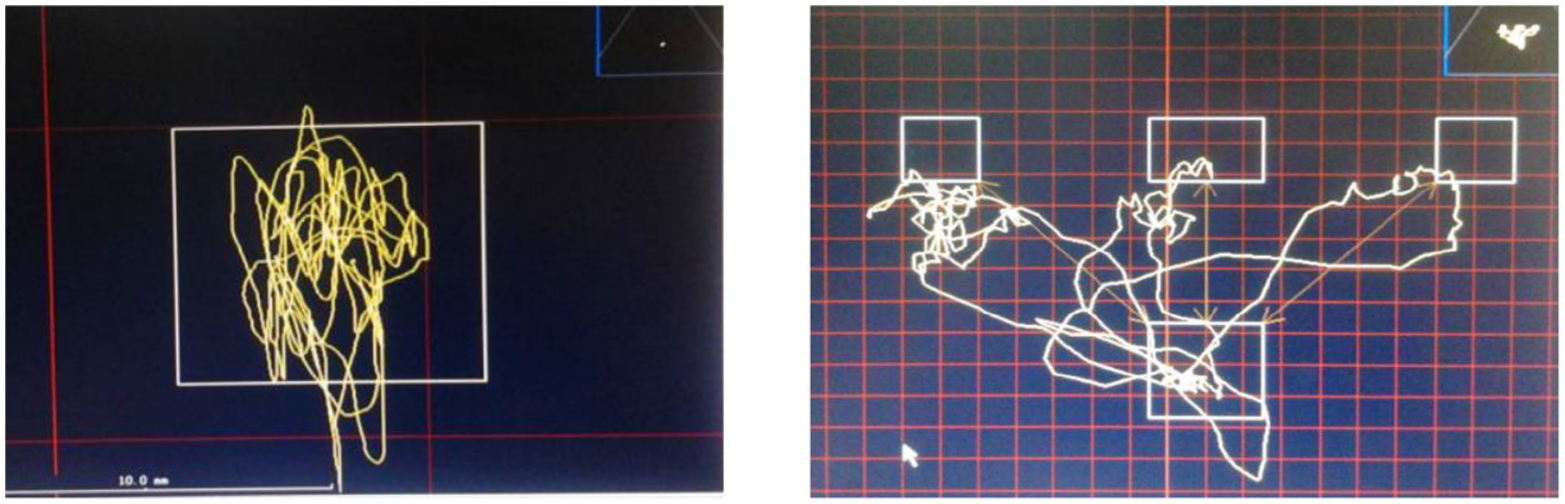
Participants’ static (left) and dynamic (right) test center of pressure trajectory diagrams.

For the static balance ability test, participants stood with both feet on the platform and were tested first with eyes open and then with eyes closed, recording the body sway within 30 s. The measurement indicator was the distance of movement of the centre of pressure during standing with eyes open and closed (left and right or front and back) (mm), with a shorter distance indicating better static balance ability (Figure 2, left side). Following this, the dynamic balance ability test was conducted where participants, without moving their feet, adjusted their body posture to reach three target positions indicated on a computer screen—left, right, and directly in front—and then immediately returned to the original test point (Figure 2, right side). The measurement indicators were the total length of displacement of the centre of pressure (mm) and the speed of movement of the centre of pressure (left and right or front and back) (mm/s). The shorter the distance the center of pressure moves along the entire path, the shorter the time taken to complete the test, and the lower the speed, the stronger the participants’ ability to precisely control their body posture, indicating better dynamic balance ability (28). Throughout the balance ability testing process, an experimenter was present to protect the participants and prevent falls. While the Good Balance testing device can automatically measure standing time, a stopwatch was used for the one-leg standing test to ensure real-time observation and consistency with previous studies. After a rest of about 10 min, the one-leg standing test with eyes open and closed was conducted, with a 1-min interval between the two testing methods, and each was measured three times with a 30-s rest in between, taking the average value.

### Statistical analysis

Data from the three measurements for both groups of participants were processed using SPSS 20.0 to calculate the mean ± standard deviation. Shapiro–Wilk test was used to check the normality of the measurement data. A two-way ANOVA was used to analyze the main effects of group (2) and time (3), as well as the interaction effect between group and time. If an interaction effect was present, separate effects of time or group were analyzed; if no interaction effect was found, the main effects of group or time were analyzed (29, 30). Post-hoc comparisons within groups at different time points were conducted using the LSD method, with a significance level of *α* = 0.05.

## Results

**Figure 3:**
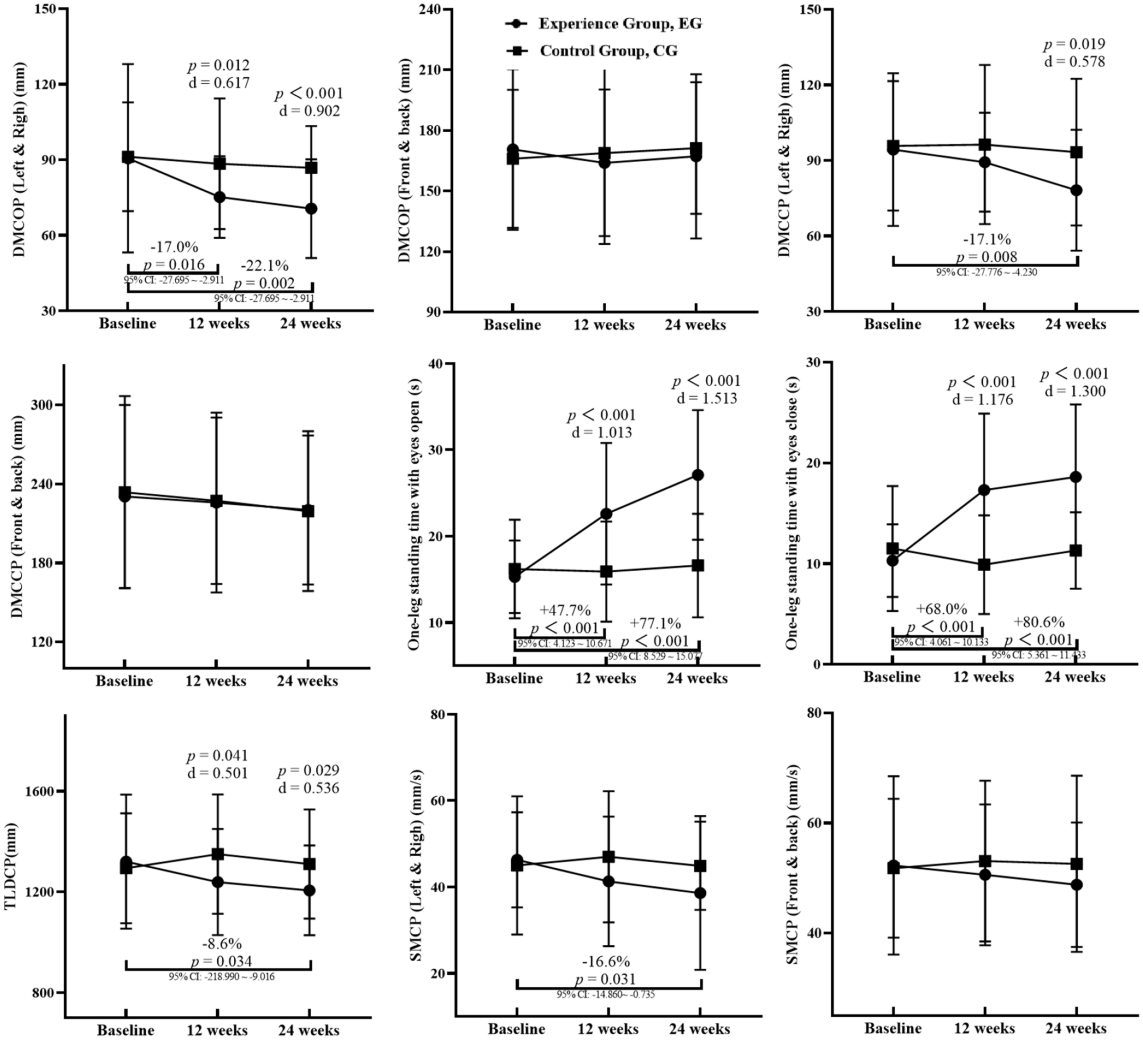
Participants’ balance ability test results. DMCOP, distance of movement of the centre of pressure during standing with eyes open; DMCCP, distance of movement of the centre of pressure during standing with eyes closed; TLDCP, total length of displacement of the centre of pressure; SMCP: Speed of movement of the centre of pressure; d: Cohen’s d, Comparison of the same-time between the groups; Comparison of the EG at Week 12 or Week 24 with Baseline.

The results of the balance ability tests for the two groups of participants are presented in Figure 3. A Shapiro–Wilk test indicated that the measurement data in this study followed a normal distribution. At baseline, there were no statistically significant differences in all measured data (*p* > 0.05). The main effects of the two-way ANOVA revealed that there were interaction effects between group and time for one-leg standing time with eyes open (*η*^2^ = 0.128) and closed (*η*^2^ = 0.115) (*p* < 0.001). Further analysis found that there were separate main effects for group (*η*^2^ = 0.158 for eyes open, *η*^2^ = 0.135 for eyes closed) and time (*η*^2^ = 0.137 for eyes open, *η*^2^ = 0.083 for eyes closed) (*p* < 0.001). For other indicators, there was no interaction effect between group and time (*p* > 0.05), but there were main effects for group in distance of movement of the centre of pressure during standing with eyes open (left and right) (*η*^2^ = 0.043, *p* = 0.003), distance of movement of the centre of pressure during standing with eyes closed (left and right) (*η*^2^ = 0.021, *p* = 0.034), total length of displacement of the centre of pressure (*η*^2^ = 0.020, *p* = 0.039), and speed of movement of the centre of pressure (left and right) (*η*^2^ = 0.019, *p* = 0.048). There was also a time main effect for distance of movement of the centre of pressure during standing with eyes open (left and right) (*η*^2^ = 0.046, *p* = 0.008). The specific analysis is as follows:

Between-group comparisons: At the 12th week, compared to the control group, the experimental group showed a decrease in distance of movement of the centre of pressure during standing with eyes open (left and right) (*p* = 0.012, Cohen’s d = 0.617) and total length of displacement of the centre of pressure (*p* = 0.041, Cohen’s d = 0.501). The one-leg standing time with eyes open (Cohen’s *d* = 1.013) and closed (Cohen’s d = 1.176) increased (*p* < 0.001). At the 24th week, compared to the control group, the experimental group showed a decrease in distance of movement of the centre of pressure during standing with eyes open (left and right) (*p* < 0.001, Cohen’s d = 0.902), distance of movement of the centre of pressure during standing with eyes closed (left and right) (*p* = 0.019, Cohen’s *d* = 0.578), and total length of displacement of the centre of pressure (*p* = 0.029, Cohen’s d = 0.536). The one-leg standing time with eyes open (Cohen’s d = 1.513) and closed (Cohen’s *d* = 1.300) increased (*p* < 0.001).

Within-group comparisons: There were no statistically significant differences between different time points in the control group (*p* > 0.05). Compared to baseline in the experimental group, the distance of movement of the centre of pressure during standing with eyes open (left and right) decreased by 17.0% at the 12th week and 22.1% at the 24th week (95% CI: −27.695 ~ −2.911, *p* = 0.016; 95% CI: −32.392 ~ −7.608, *p* = 0.002). The distance of movement of the centre of pressure during standing with eyes closed (left and right), total length of displacement of the centre of pressure, and speed of movement of the centre of pressure (left and right) decreased by 17.1, 8.6, and 16.6% at the 24th week (95% CI: −27.776 ~ −4.230, *p* = 0.008; 95% CI: −218.990 ~ −9.016, *p* = 0.034; 95% CI: −14.860 ~ −0.735, *p* = 0.031). The one-leg standing time with eyes open and closed times increased by 47.7 and 68.0% at the 12th week (95% CI: 4.123 ~ 10.671, *p* < 0.001; 95% CI: 4.061 ~ 10.133, *p* < 0.001), and by 77.1 and 80.6% at the 24th week (95% CI: 8.529 ~ 15.077, *p* < 0.001; 95% CI: 5.361 ~ 11.433, *p* < 0.001). Compared to the 12th week, the one-leg standing time with eyes open increased by 19.9% at the 24th week (95% CI: 1.132 ~ 7.680, *p* = 0.009).

## Discussion

This study conducted a 24-week Wuqinxi intervention for older women with a history of falls to explore the changes in static and dynamic balance abilities. We confirmed part of our research hypothesis: Wuqinxi exercise increased the static and dynamic balance abilities in both left–right directions for older women with a history of falls. Compared to 12 weeks, 24 weeks of Wuqinxi exercise further enhanced the static balance ability in the open-eye state.

The current study on the impact of Wuqinxi exercise on the balance ability of the older mainly measures the changes in the one-leg standing time with eyes open and closed for participants. Sixteen weeks of Wuqinxi exercise can significantly increase the one-leg standing time with eyes closed for older women (24). Twenty weeks of Wuqinxi exercise significantly increased both the open-eye and closed-eye one-leg standing time for middle-aged and older women (25). This study extended the intervention time to 24 weeks, and the participants’ one-leg standing time with eyes open and closed had significantly increased at 12 weeks, consistent with previous studies (24, 25), and the effect was better at 24 weeks, indicating that with the increase in the duration of Wuqinxi practice, the effect of improving static balance ability is more obvious.

This study used the Good Balance balance testing device to analyze the changes in the participants’ static and dynamic balance abilities in the left–right and front-back directions, refining the specific direction of balance ability changes. We found an interesting conclusion: Wuqinxi exercise could not improve the static and dynamic balance abilities in the front-back direction for the older female participants. This is consistent with the study by Wang et al. (21), where the author conducted a 24-week Wuqinxi exercise for older women without a history of falls, showing an increase in static and dynamic balance abilities in the left–right direction. This study involved older women with a history of falls, further expanding the theory of the impact of Wuqinxi exercise on human balance ability. It is well known that the front-back direction is an area that the older often exercise in daily life, such as walking, jogging, and climbing stairs, while the left–right direction activities are relatively less, and when conducting specific exercise interventions, if there are left–right directional movement exercises, it is easier to improve the left–right directional balance ability compared to the front-back direction (31). A video analysis of falls in the older found that the disruption of balance in the left–right direction is an important cause of falls, and compared to the front-back direction, the stability in the left–right direction is more easily disrupted (32). Therefore, improving the balance control ability of the older in the left–right area is important.

This study found that 12 weeks of Wuqinxi exercise had already improved the participants’ static balance ability in the left–right direction, and the effect was better at 24 weeks. As for the improvement of dynamic balance ability, significant effects were only observed at 24 weeks. Previous studies on middle-aged women for 48 weeks of Wuqinxi exercise, measuring the Berg scale score at baseline, 24 weeks, and 48 weeks, found that with the increase in exercise time, the Berg scale score showed an increasing trend (23). For older adults with knee osteoarthritis, a condition associated with impaired balance (33), 24 weeks of Wuqinxi exercise improved static balance ability and reduced dynamic fall index (34). For moderate Parkinson’s patients, 12 weeks of Wuqinxi exercise improved dynamic balance ability (35). This study to some extent confirmed the previous studies (23, 34), but there are also differences with the previous studies (35). This may be related to the different participant groups, this study is for older women with a history of falls, while the previous studies included knee osteoarthritis patients (34) and moderate Parkinson’s patients (35), as well as the different means of measuring balance ability.

Analyzing the reasons for improving human balance ability from the characteristics of Wuqinxi exercise movements. First, participants are required to imitate animal behavior. First, the Tiger Play: imitating the tiger’s pounce action, participants are required to make quick and powerful turning and pouncing movements. This kind of movement helps to improve reaction speed and body coordination (21–25). Second, the Deer Play: imitating the deer’s running, through continuous jumping and turning, it enhances lower limb strength and flexibility. In addition, by imitating the deer’s horn-butting action, participants need to maintain balance on one leg while performing upper body resistance movements, which helps to improve the stability of standing on one leg (34). Third, the Bear Play: imitating the bear’s sway, through the body’s left and right swaying, it enhances body stability and sense of balance. Fourth, the Ape Play: imitating the ape’s arm-raising action, participants are required to extend and retract their upper limbs while maintaining balance, enhancing upper limb strength and coordination (35). Fifth, the Bird Play: imitating the bird’s wings spreading and stretching, through large-amplitude limb stretching, it enhances body flexibility and sense of balance.

Secondly, participants are required to constantly change the body’s support surface and center of gravity. During the practice of Wuqinxi, participants need to constantly change the body’s support surface and center of gravity height. For example, in the Ape Lift and Bird Stretch movements, participants need to maintain balance through standing on one leg and raising the leg backward. This kind of exercise helps to enhance the strength and endurance of lower limb muscles and improve body stability. At the same time, many movements in Wuqinxi require participants to alternately use the left and right legs for standing on one leg, which helps to improve the balance and coordination of the muscles on both sides. This is particularly important for the older because they often have differences in balance ability in the left–right direction (25).

Finally, participants are required to combine dynamic and static balance during practice. Wuqinxi not only includes static balance exercises (such as standing on one leg) but also includes dynamic balance exercises (such as continuous jumping and turning) (24). This combination helps to comprehensively improve the balance ability of the older, enabling them to better cope with various dynamic and static balance challenges in daily life. Through the practice of these movements, Wuqinxi can not only enhance the muscle strength and flexibility of the older but also improve their reaction speed and coordination, thereby significantly improving their balance ability. This is of great significance for preventing falls and improving the quality of life of the older.

This study has certain limitations. First, geographical limitations. The study participants all come from the same area, which may affect the generality of the results. In future studies, participants from different regions should be recruited to improve the generality and representativeness of the study results; second, the single mode of exercise. It has not been compared with other modes of exercise, and the effect of Wuqinxi relative to other exercises cannot be evaluated. In future studies, other modes of exercise (such as Tai Chi, aerobic fitness exercises, etc.) should be set as control groups to compare the effects of different modes of exercise on balance ability; finally, This study did not monitor post-intervention fall incidence or assess neuromuscular responses (e.g., proprioception, muscle activation patterns), which limits the generalizability of findings to fall prevention outcomes. These issues are expected to be resolved in subsequent studies.

## Conclusion

The 24-week Wuqinxi exercise significantly improved the static and dynamic balance abilities in the left–right direction for older women with a history of falls. Compared to 12 weeks, a longer duration of Wuqinxi practice further enhanced their static balance ability in the open-eye state. This finding indicates that Wuqinxi, as a gentle and effective form of exercise, can significantly improve the balance ability of the older and reduce the risk of falls.

## Data Availability

The original contributions presented in the study are included in the article/supplementary material, further inquiries can be directed to the corresponding author.
